# Active Dual‐Protein Coating Assisted by Stepwise Protein–Protein Interactions Assembly Reduces Thrombosis and Infection

**DOI:** 10.1002/advs.202310259

**Published:** 2024-02-29

**Authors:** Wentai Zhang, Jiangling Zhang, Fangkun Hu, Wenxuan Wang, Zeyu Du, You Ke, Qing Ma, Xiaohui Mou, Jing Lu, Zhilu Yang

**Affiliations:** ^1^ Dongguan Key Laboratory of Smart Biomaterials and Regenerative Medicine The Tenth Affiliated Hospital Southern Medical University Dongguan Guangdong 523000 China; ^2^ School of Materials Science and Engineering Key Lab of Advanced Technology for Materials of Education Ministry Southwest Jiaotong University Chengdu 610031 China; ^3^ Department of Anesthesiology Sichuan Provincial People's Hospital University of Electronic Science and Technology of China Chengdu Sichuan 610072 China

**Keywords:** active protein coating, antibacterial, antithrombosis, central venous catheters, electrostatic assembly

## Abstract

Universal protein coatings have recently gained wide interest in medical applications due to their biocompatibility and ease of fabrication. However, the challenge persists in protein activity preservation, significantly complicating the functional design of these coatings. Herein, an active dual‐protein surface engineering strategy assisted by a facile stepwise protein–protein interactions assembly (SPPIA) method for catheters to reduce clot formation and infection is proposed. This strategy is realized first by the partial oxidation of bovine serum albumin (BSA) and lysozyme (LZM) for creating stable nucleation platforms via hydrophobic interaction, followed by the assembly of nonoxidized BSA (pI, the isoelectric point, ≈4.7) and LZM (pI ≈11) through electrostatic interaction owing to their opposite charge under neutral conditions. The SPPIA method effectively preserves the conformation and functionality of both BSA and LZM, thus endowing the resultant coating with potent antithrombotic and bactericidal properties. Furthermore, the stable nucleation platform ensures the adhesion and durability of the coating, resisting thrombosis and bacterial proliferation even after 15 days of PBS immersion. Overall, the SPPIA approach not only provides a new strategy for the fabrication of active protein coatings but also shows promise for the surface engineering technology of catheters.

## Graphical abstract

This study proposes a stepwise protein–protein interactions assembly strategy to fabricate an active dual‐protein coating, balancing the deposition efficiency and preservation of protein activity. The findings suggest that the assembled bovine serum albumin (BSA) and lysozyme (LZM) synergistically endow the modified surfaces with enhanced antithrombosis and antibacterial properties, exhibiting application potential for central venous catheters.

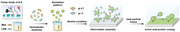

## 1 Introduction

Proteins are essential components in organisms and function through diverse assembly forms.^[^ ^1^ ^]^ Protein films, in particular, are a common assembly form in various organisms. Examples include the S‐layer protein of bacteria and the hydrophobin film produced by fungi, both of which are critical in structural integrity and biological functions.^[^ ^2^ ^]^ Inspired by these functional natural films, a range of protein‐based coatings has been developed in practical applications, such as food packaging,^[^ ^3^ ^]^ textiles,^[^ ^4^ ^]^ and particularly in biomedical devices.^[^ ^5^ ^]^ However, despite the recent advancements in supramolecular assembly strategies,^[^ ^6^ ^]^ the realization and design of biological functionalities for protein coatings still present a significant challenge.

Universal amyloid‐like protein coatings have recently gained increasing interest because of their durability and ease of fabrication.^[^ ^7^ ^]^ Meanwhile, these coatings are particularly promising in biomedical applications for their certain biological functionalities.^[^ ^8^ ^]^ For instance, the amyloid‐like lysozyme (LZM) coating exhibits potent antimicrobial properties and good blood compatibility,^[^ ^9^ ^]^ showing potential for catheter applications. However, it is important to note that such bactericidal effects are largely attributed to specific surface properties (e.g., positive charged and hydrophobic residues on the surface),^[^ ^10^ ^]^ which are realized by the specific reaction condition (e.g., acidic condition). Consequently, the bactericidal effect was absent in the amyloid‐like LZM coating fabricated under neutral conditions.^[^ ^11^ ^]^ Typically, the formation of amyloid‐like protein coatings relies on a complete phase transition process induced by tris(2‐carboxyethyl)phosphine.^[^ ^12^ ^]^ This process involves the full cleavage of disulfide (S─S) bonds and often leads to a significant decrease or even total loss of protein activity.^[^ ^13^ ^]^ Therefore, achieving specific functionalities in these coatings requires precise control of the reaction condition to obtain desired surface properties, making the functional design of amyloid‐like protein coatings a particular challenge.

The hypothesis is that by preserving the activity of native proteins in coatings, the functionalities of protein coatings can be more predictable and easier to design, thereby meeting a broader range of application requirements. Recently, several strategies have been explored for maintaining the activity of protein coatings. For instance, Chen et al. successfully regulated the self‐assembly of salmon calcitonin to create active protein materials by fine‐tuning the protein–protein interactions through pH adjusting.^[^ ^14^ ^]^ Similarly, inspired by chemoselective reactions, Yang et al. developed a technology to selectively cleave S─S bonds away from protein active sites, effectively minimizing their activity loss.^[^ ^15^ ^]^ Although these methods have shown efficacy in preserving protein activity, their application is generally limited to single‐protein systems. The fine‐tuning of protein assembly requires a tailored approach based on the intrinsic properties of each specific protein (e.g., orientation, complexation constants, charge number, stimulus responsiveness).^[^ ^8^ ^]^ Importantly, the functionalities offered by single‐protein coatings are often insufficient in meeting the complex biological demands of clinical applications.^[^ ^16^ ^]^ Therefore, developing an innovative technology that facilitates the deposition of multiple proteins and the preservation of their activities holds great promise for enhancing the potential of protein coatings in biomedical applications.

Central venous catheters (CVCs) play an instrumental role in clinical settings, facilitating various medical procedures such as medication delivery, hemodialysis, and parenteral nutrition support.^[^ ^17^ ^]^ However, their prolonged indwelling is commonly associated with a high incidence of thrombosis and infection.^[^ ^18^ ^]^ Currently, the primary preventive approach involves the systemic administration of antibiotics and anticoagulants during CVCs indwelling. Nonetheless, their excessive use often leads to severe adverse effects, such as bacterial antibiotic resistance and uncontrolled bleeding.^[^ ^19^ ^]^ Consequently, novel strategies are urgently needed to effectively and simultaneously address thrombosis and infection. Herein, we propose an active dual‐protein coating assisted by a facile stepwise protein–protein interactions assembly (SPPIA) method for the application of CVCs. This SPPIA approach is realized by the partial oxidization of two proteins with opposing net charges, namely LZM (pI, the isoelectric point, ≈11) and bovine serum albumin (BSA, pI ≈4.7), under neutral conditions. Unlike amyloid‐like coatings that undergo full‐phase transition, the partial oxidization of BSA and LZM using sodium persulfate (SP) creates solely stable nucleation platforms through hydrophobic interaction. Subsequently, the residual nonoxidized BSA and LZM are electrostatically assembled onto these platforms under neutral conditions, promoting their growth and inter‐particle fusion, finally resulting in the formation of the active protein coating. The neutral condition and electrostatic interaction both help in preserving the conformation, as well as the activity and functionality of both proteins. As a result. the bioinert BSA contributes to antithrombosis by preventing protein and cell adhesion,^[^ ^20^ ^]^ while LZM offers bactericidal properties through its enzymatic activity.^[^ ^21^ ^]^ Moreover, the stable nucleation platform of amyloid‐like proteins ensures the adhesion and durability of the coating. We believe that this SPPIA method not only introduces a novel approach for bioactive protein assembly but also presents a promising surface engineering technology for vascular catheters, addressing critical clinical needs.

## 2 Results and Discussion

### 2.1 Formation Mechanism of Active Dual‐Protein Coating

**Figure 1:**
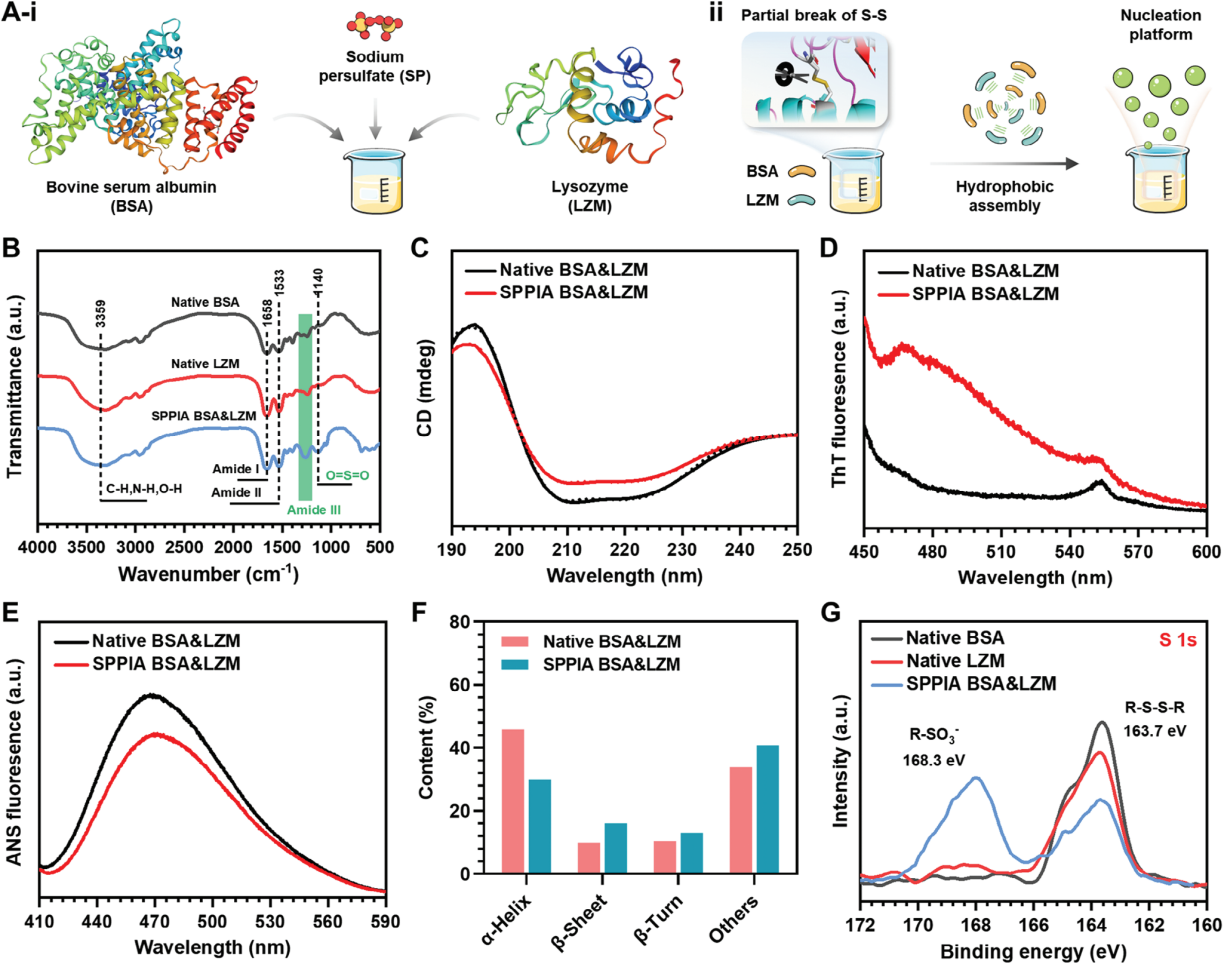
Nucleation platform formation via phase transition‐induced hydrophobic interactions. A‐i) Partial oxidization of BSA and LZM using SP with one‐pot method. A‐ii) Formation of nucleation platforms through hydrophobic assembly among BSA and LZM following the S─S bonds breakdown. B) FTIR spectra of native BSA, native LZM, and SPPIA BSA&LZM. C) CD, D) ThT fluorescence, and E) ANS fluorescence spectra of native and SPPIA BSA&LZM. F) Quantitative analysis of structural content in native and SPPIA BSA&LZM according to the CD analysis. G) High‐resolution S 1s spectra of native BSA, native LZM, and SPPIA BSA&LZM.

The active dual‐protein coating was fabricated through a stepwise process. First, the BSA and LZM were mixed in an appropriate oxidizing environment, optimized by the adjustment of SP concentration (**Figure** **1**A‐i). This environment facilitated the phase transition and amyloid‐like aggregation of solely partial proteins, resulting in the formation of nanoparticle‐like aggregates in the solution, which serve as nucleation platforms (Figure 1A‐ii). It has been reported that S─S bonds in proteins play a critical role in their conformation change,^[^ ^22^ ^]^ which are widely utilized in creating phase‐transited protein coatings. In our study, the phase transition of BSA and LZM was also facilitated by the disruption of S─S bonds. The strong oxidants could break down the S─S bonds in the cystine sequences and easily transfer the resultant sulfhydryl (─SH) group into sulfonic acid (─SO_3_H) groups.^[^ ^23^ ^]^ This was evidenced by the Fourier‐transform infrared spectroscopy (FTIR) analysis, where the significant shift of Amide III peak for the SPPIA BSA&LZM was observed compared to the native BSA and LZM (Figure 1B), suggesting the changes in amino acid residues. Moreover, the presence of a characteristic peak for O═S═O (at 1140 cm^−1^) in the SPPIA BSA&LZM further confirmed this conclusion.

After the breakdown of S─S bonds, conformational changes and aggregation in BSA and LZM were initiated (Figure 1C–E). The Far‐UV circular dichroism (CD) spectrum of SPPIA BSA&LZM revealed a partial loss of their α helix structure compared to their native state, as evidenced by the shift of characteristic peaks at 208 and 222 nm (Figure 1C). Meanwhile, thioflavin T (ThT) fluorescence spectra demonstrated a notable increase in the β‐sheet structure content in SPPIA BSA&LZM (Figure 1D). The increase in β‐sheet structure suggests protein unfolding, which exposes hydrophobic residues on their surface and subsequently induces the formation of protein aggregates.^[^ ^24^ ^]^ These hydrophobic interactions are considered the primary driving force for the formation of phase‐transited protein coatings. However, the measurement of 8‐anilino‐1‐naphthalenesulfonate acid (ANS) binding indicated a slight reduction in the hydrophobicity of SPPIA BSA&LZM (Figure 1E). Unlike fully phase‐transited protein coatings, our method involved only partial oxidation of proteins to create hydrophobic cores, serving as nucleation platforms for the following nonoxidized protein assembly. This is evidenced by the largely preserved α helix structure (Figure 1F) and the similar proportion of S─S and R─SO_3_^−^ bonds (Figure 1G) in SPPIA BSA&LZM. Therefore, a large amount of residual non‐oxidized proteins could rapidly envelop these hydrophobic cores via hydrophobic interactions, then exposing their hydrophilic sequences externally. Therefore, a slightly reduced hydrophobicity in SPPIA BSA&LZM was observed in this work.

**Figure 2:**
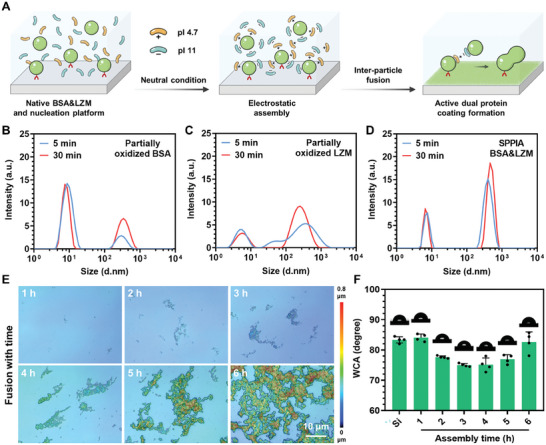
Active dual protein coating formation through electrostatic interactions. A) Schematic illustration of the self‐assembly of oppositely charged BSA and LZM onto nucleation platforms, followed by their inter‐particle fusion, which is driven by electrostatic interactions. Particle size distributions in the solutions of B) BSA, C) LZM, and (D) BSA&LZM after 5 and 30 min of partial oxidation. E) Evolution of 3D surface morphology and F) WCA of SPPIA BSA&LZM coated substrates with various deposition times (*n* = 4, mean + SD).

Following the formation of nucleation platforms, its growth, and inter‐particle fusion are essential for the development of coatings. Unlike the formation of fully phase‐transited protein coatings, which predominantly rely on hydrophobic interactions, our active dual‐protein coating formation was assisted by two distinct forces. Initially, as descripted above, the nucleation platform in our active dual‐protein coating was formed through a protein phase transition and the resultant hydrophobicity‐induced aggregation. After this, the subsequent assembly of nonoxidized proteins was driven by electrostatic interactions, facilitated by the distinct pI of BSA and LZM (**Figure** **2**A). The pI values of proteins dictate their surface charge in solutions of varying pH. In a neutral environment, native BSA (pI ≈ 4.7) and LZM (pI ≈ 11) display opposite surface charges, enabling the electrostatic interaction‐driven assembly in our system. As observed in Figure 2B,C, the particle sizes of individual BSA and LZM exhibit a slight increase with progressing partial oxidation, suggesting the nucleation platform formation through hydrophobic interactions. Notably, the particle size of the partially oxidized BSA&LZM was significantly larger than that of individual partially oxidized BSA and LZM (Figure 2D). Specifically, after 5 min of oxidization, the proportion of particles in the hundreds of nanometers range for SPPIA BSA&LZM was more than double that of particles in the several nanometers range, approximately corresponding to the initial protein size. As oxidation continues, the particle size further increases. However, due to the limitations in detection range, the particle size after 30 min of oxidation could not be accurately determined. These results indicated the successful participation of electrostatic interactions in particle fusion and coating formation.

To further clarify the formation mechanism of the SPPIA BSA&LZM coating, we investigated the particle fusion on the substrate surface. The surface morphology of the SPPIA BSA&LZM coating with varied deposition times was analyzed using a 3D profilometer. As depicted in Figure 2E, an increase in deposition time led to a rise in the number of particles on the surface. However, these particles were not uniformly distributed. Instead, they exhibited a tendency to cluster, probably due to the hydrophobic and/or electrostatic interactions among them. Importantly, with prolonged deposition time, the fusion of particles became increasingly apparent. After 6 h of deposition, a continuous structure composed of protein particles was observed, confirming the effective coating‐forming capability of our system. The water contact angle (WCA) of coated surfaces after different deposition times was also measured. As illustrated in Figure 2F, the WCA initially decreased from 84° to 76° during the first 4 h of deposition. This decrease aligns well with our proposed nucleation formation process. Nonoxidized proteins envelop the unfolding hydrophobic protein core, resulting in a rise in surface hydrophilicity due to the increased exposure of hydrophilic sequences. However, the subsequent increase in WCA may be caused by the increasing amount of nonoxidized proteins electrostatically assembled on the surface and the balance of exposed hydrophilic and hydrophobic sequences.

### 2.2 Fabrication of SPPIA BSA&LZM Coating on CVCs

**Figure 3:**
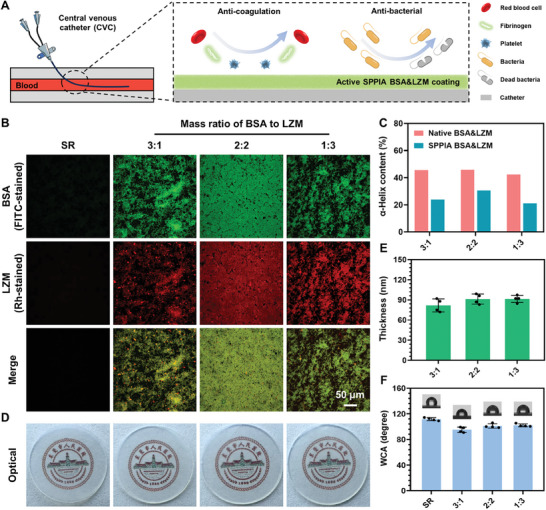
Fabrication and characterization of SPPIA BSA&LZM coating on the SR substrate for CVCs application. A) Schematic illustration of the SPPIA BSA&LZM coated catheter with anticoagulation and antibacterial properties. B) Fluorescence images of SPPIA BSA&LZM coated SR substrates with different ratios of BSA to LZM. BSA and LZM were prestained with FITC and Rhodamine, respectively. C) Quantitative analysis of α‐Helix structure content in various SPPIA BSA&LZM coatings according to the CD analysis. D) Photographs of bare and SPPIA BSA&LZM coated SR substrates. E) Thickness of SPPIA BSA&LZM coatings on the SR substrate (*n* = 4, mean ± SD). F) WCA of SPPIA BSA&LZM coated SR substrates (*n* = 4, mean ± SD).

After understanding the formation mechanism of the SPPIA BSA&LZM coating, we further applied this coating to CVCs to evaluate the bioactivity maintenance of the assembled proteins on their surfaces. Prolonged use of CVCs is commonly associated with a high incidence of thrombosis and infection. In this context, the assembled BSA is expected to reduce the adhesion of proteins, cells, and bacteria, thereby contributing to the reduction of thrombosis and infection risks (**Figure** **3**A). In addition, LZM is expected to enhance the bactericidal activity on the surface due to its enzymatic action. The synergistic effects of BSA and LZM render this coating particularly advantageous for CVCs. Therefore, this application serves as an ideal model to demonstrate the practical benefits of our strategy in biomedical devices.

To achieve optimal antithrombosis and anti‐infection capabilities, three different mass ratios of BSA to LZM were performed for the active dual‐protein coating fabrication on silicon rubber (SR), which is a commonly used material for CVCs. Additionally, the uniformity and integrity of the coating are crucial for its effectiveness. We found that a single deposition period exceeding 6 h improved the coverage of the coating. However, this also led to the formation of oversized particles, which detrimentally affected the uniformity of the coating. Therefore, to ensure the uniformity and full coverage of our protein coating, the deposition process was conducted three times, each lasting 6 h. After each deposition, the coated SR was subjected to ultrasonic cleaning to remove unstably adhered particles. As depicted in Figure S1 (Supporting Information), the coatings with varying mass ratios were successfully and uniformly deposited on the SR surfaces, despite the presence of some micrometer‐sized particles. The FTIR analysis further confirmed the successful deposition of these coatings, as evidenced by the presence of O ═ S = O peak and a noticeable shift in the Amide III peak (Figure S2, Supporting Information).

The integration of the dual proteins in coatings was assessed by prestaining them before coating fabrication. To ensure the fluorescence intensity, the deposition process was performed once. As shown in **Figure** **3**B, both the BSA and LZM were effectively integrated into the coating and well blended with each other, confirming the successful fabrication of our dual‐protein coatings. Notably, the most effective assembly was observed with a mass ratio of 2:2. This can be attributed to the similar quantities of surface charges and their opposite nature in BSA and LZM at this ratio, which enhances electrostatic interactions between them. Moreover, increased electrostatic interactions are beneficial for the preservation of the α helix structure of proteins in the coating (Figure 3C; Figures S3–S5, Supporting Information). Notably, the α helix structure content in the SPPIA BSA&LZM coating with 2:2 mass ratio was 30.6%, which is higher compared to 23.9% and 21.1% in the 3:1 and 1:3 mass ratios, respectively. It has been reported that the preservation of the α‐helix structure in proteins is beneficial for maintaining their biofunctionalities. Consequently, the SPPIA BSA&LZM coating with a 2:2 mass ratio is likely to offer an optimized synergistic effect of the two proteins.^[^ ^25^ ^]^ Additionally, it is important to note that the coatings exhibited high optical transparency without the intrinsic color (Figure 3D). This feature can facilitate the observation of the fluid flow or any potential blockages within CVCs, thereby ensuring easier monitoring of their patency. Furthermore, the thickness of the coating was consistently maintained between 80 and 90 nm, which was not significantly influenced by the mass ratio of BSA to LZM (Figure 3E). Similarly, all the samples demonstrated comparable WCA (≈100°) (Figure 3F). These characteristics indicate the uniformity of the physical properties of the coating across varying compositions.

### 2.3 Antibacterial Efficacy of SPPIA BSA&LZM Coating

**Figure 4:**
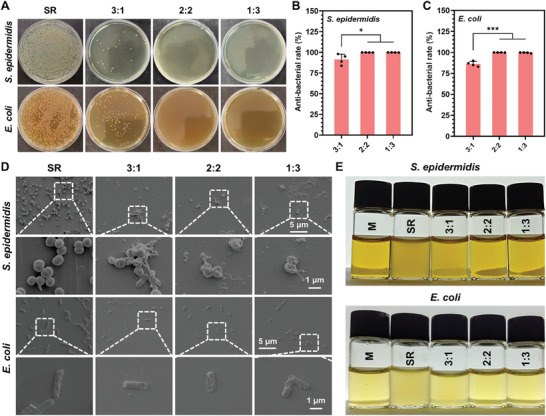
Antibacterial efficacy of SPPIA BSA&LZM coatings against *S. epidermidis* and *E. coli*. A) Typical colonies of *S. epidermidis* and *E. coli* after 24 h incubation on the SPPIA BSA&LZM coated SR substrates with different mass ratios of BSA to LZM. Quantitative analysis of the antibacterial rate against B) *S. epidermidis* and C) *E. coli*, calculated from colony counts (*n* = 4, mean + SD). D) Representative SEM images of bacteria adhered on the SPPIA BSA&LZM coated SR substrates. E) Photographs of bacterial solutions of *S. epidermidis* and *E. coli* after 24 h incubation on the SPPIA BSA&LZM coated SR substrates. Statistical analysis was performed using one‐way ANOVA with ^*^ *p* < 0.05 and ^***^ *p* < 0.001.

To assess the bioactivity preservation of proteins in our SPPIA dual‐protein coating, we first examined its antibacterial efficacy against gram‐negative *Escherichia coli* (*E. coli*) and gram‐positive *Staphylococcus epidermidis* (*S. epidermidis*) in solid culture. It was observed that all the SPPIA dual‐protein coatings endowed the SR with the ability to resist *E. coli* and *S. epidermidis* (**Figure** **4**A). Furthermore, the increase in LZM content in the coating led to enhanced antibacterial properties. Specifically, coatings with 2:2 and 1:3 mass ratios of BSA to LZM exhibited ≈100% antibacterial rate for both *E. coli* and *S. epidermidis* (Figure 4B,C). The coating with 3:1 mass ratio of BSA to LZM, while slightly less effective, still achieved antibacterial rates of 91.45% against *S. epidermidis* and 86.25% against *E. coli*. The antibacterial efficacy of our coatings could be attributed to the antiadhesive properties of BSA and the bactericidal properties of LZM. BSA inhibits bacterial adhesion through steric repulsion and low surface interaction energy.^[^ ^26^ ^]^ Meanwhile, the ─SO_3_H groups on the surface are known to reduce bacterial adhesion due to their negative charge, creating a repulsive force against the negatively charged bacterial membranes.^[^ ^27^ ^]^ Furthermore, LZM could destroy bacterial cell walls through enzymatic hydrolysis of the 1,4‐beta‐linkages between N‐acetylmuramic acid and N‐acetyl‐d‐glucosamine residues in peptidoglycan, a key component of bacterial cell walls.^[^ ^28^ ^]^ While the effectiveness of this bactericidal property largely depends on the bioactivity of LZM. To further validate the bactericidal properties of our coating, we examined the state of bacteria on the sample surface and the sterilization efficacy in liquid culture. As depicted in Figure 4D, both *S. epidermidis* and *E. coli* showed massive proliferation on uncoated SR. In contrast, bacteria cultured on the SPPIA BSA&LZM coating exhibited membrane rupture. Similar results were observed in liquid culture. As shown in Figure 4E and Figure S6 (Supporting Information), ≈100% of both *S. epidermidis* and *E. coli* were eliminated by the SPPIA BSA&LZM coating with 2:2 and 1:3 mass ratios, demonstrating the potent bactericidal properties and the effective bioactivity preservation of LZM in our coating. To confirm the biocompatibility of our coatings, the viability of human umbilical vein endothelial cells (HUVECs) was assessed in the extracts of coated SR substrates (Figure S7, Supporting Information). After 24 and 72 h incubation, no cell toxicity was observed, indicating the cell compatibility of our coatings.

### 2.4 Antithrombosis Property of SPPIA BSA&LZM Coating

**Figure 5:**
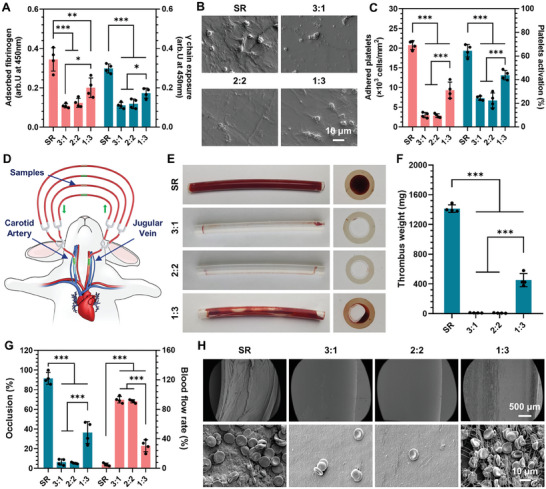
Antithrombogenic properties of SPPIA BSA&LZM coatings by in vitro and ex vivo blood compatibility tests. A) Quantitation of Fg adsorption and γ chain exposure on SPPIA BSA&LZM coated SR substrates with different mass ratios of BSA to LZM (*n* = 4, mean ± SD). B) SEM images of the adhered platelets on the coated SR substrates. C) Quantitation of platelet adhesion and activation on the coated SR substrates determined from SEM images (*n* = 4, mean ± SD). D) Schematic illustration of rabbit AV shunt model for the ex vivo blood compatibility test. E) Photographs of side and cross‐sectional views of bare and coated catheters after 2 h circulation. F) Measurement of thrombus weight formed in the bare and coated catheters after circulation (*n* = 4, mean ± SD). G) Occlusion ratio and blood flow rate in bare and coated catheters after circulation (*n* = 4, mean ± SD). H) Representative SEM images of the lumen side of bare and coated catheters after circulation. Statistical analysis was performed using one‐way ANOVA with ^*^ *p* < 0.05, ^**^ *p* < 0.01 and ^***^ *p* < 0.001.

Thrombosis is a major concern in the application of CVCs, primarily triggered by the adhesion and activation of fibrinogen (Fg) and platelet.^[^ ^29^ ^]^ To evaluate the antithrombotic efficacy of our SPPIA BSA&LZM coating, we first investigated the adsorption behavior and conformation of Fg on the modified samples by exposing them to platelet‐poor plasma (PPP). As illustrated in **Figure** **5**A, the SPPIA BSA&LZM coatings significantly reduced Fg adsorption, demonstrating their antiadhesive properties. Notably, the antiadhesive properties were more effective in coatings with 3:1 and 2:2 mass ratios. However, a further decrease in BSA content, as in a lower mass ratio, weakened this antiadhesive effect, suggesting the crucial role of the BSA component in the antiadhesion capability of the coating. In addition to the adsorption of Fg, the conformation changes of absorbed Fg are equally critical for thrombosis formation. It has been reported that the γ chain of Fg significantly contributes to platelet aggregation through its interaction with the GPIIb‐IIIa receptor at the carboxy‐terminal end.^[^ ^30^ ^]^ Therefore, we assessed the activity of the γ chain in Fg adsorbed on our coatings. Similar to the Fg adsorption results, higher activities of γ chains were observed on the uncoated SR surface. In contrast, the SPPIA BSA&LZM coating largely preserved the natural conformation of the adsorbed Fg, suggesting a potential positive effect on anticoagulation. Additionally, coatings with a higher content of BSA were more effective in maintaining the natural conformation of Fg. This is consistent with the previous study indicating that Fg tends to maintain a more natural conformation in the presence of BSA.^[^ ^31^ ^]^ The results suggested that our SPPIA dual‐protein coating can not only reduce the Fg adsorption but also minimize its conformational alterations. Furthermore, we exposed the modified samples to platelet‐rich plasma (PRP) to investigate the response of platelets to the SPPIA BSA&LZM coated surfaces in the presence of Fg. It can be observed that numerous activated platelets with extending filopodia were distributed on the surface of uncoated SR (Figure 5B). While after coating with SPPIA BSA&LZM, there was a significant reduction in platelet adhesion and activation. Notably, almost no adherent platelets were observed on coatings with mass ratios of 3:1 and 2:2. This observation was further supported by the quantification of the number of adherent and activated platelets (Figure 5C). It has been reported that the conformation of adsorbed Fg plays a critical role in both platelet adhesion and activation.^[^ ^32^ ^]^ Therefore, the reduction in platelet adhesion and activation should be mainly attributed to the conformation maintenance of BSA on adsorbed Fg, as well as the antiadhesive properties of our SPPIA BSA&LZM coating. These results demonstrate the effectiveness of our SPPIA dual protein coating in preventing the adsorption and conformation changes of Fg, and the adhesion and activation of platelets.

To further investigate the antithrombogenic capabilities of our SPPIA BSA&LZM coating, we performed an ex vivo blood circulation experiment using commercially available SR catheters. The bare and modified catheters were connected to a rabbit arteriovenous (AV) shunt (Figure 5D). Notably, no anticoagulants were utilized in this experiment. After 2 h of circulation, significant thrombus formation was observed in the catheters of the SR group (Figure 5E). In contrast, catheters modified with the SPPIA BSA&LZM coating exhibited only minor clotting. Similar to the in vitro antithrombogenic results, coatings with a higher BSA content were more effective in inhibiting thrombus formation. This observation was further confirmed by quantitative analyses, including the weight of the thrombus formed in the catheters (Figure 5F), as well as the occlusion ratio and blood flow rate (Figure 5G). For instance, catheters coated with SPPIA BSA&LZM at a 2:2 mass ratio significantly reduced the thrombosis weight (1413.78 mg vs 8.87 mg) and occlusion ratio (5.35% vs 91.55%), resulting in an improved blood flow rate (91.05% vs 7.55%) compared to the SR group. Field‐emission scanning electron microscopy (SEM) images further revealed the aggregation of erythrocytes and platelets and the formation of fibrous‐like thrombus structures on the bare SR catheter. Only a small amount of red blood cells was observed on SPPIA BSA&LZM coating with mass ratios of 3:1 and 2:2, suggesting the effectiveness in preventing thrombus formation. Moreover, different SPPIA BSA&LZM coatings did not cause hemolysis (Figure S8, Supporting Information), suggesting their good blood compatibility.

**Figure 6:**
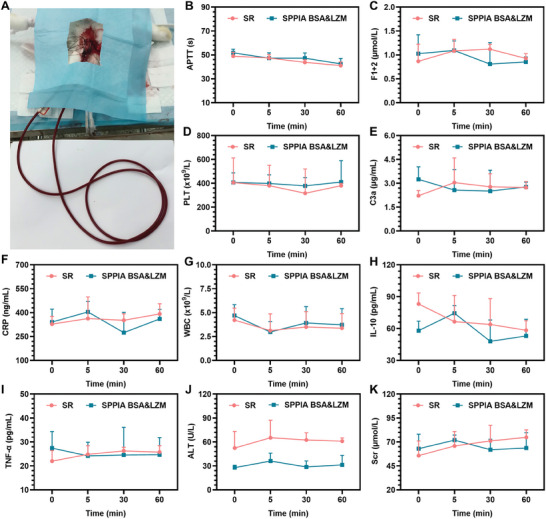
Blood analysis by ex vivo perfusion experiment. A) Ex vivo perfusion model on rabbit for blood analysis. Examination of blood parameters including B) APTT, C) F1+2 (an integral marker for prothrombin activation), D) PLT, E) C3a (a fragment produced by cleavage of C3), F) CRP, G) WBC, H) IL‐10, I) TNF‐α, J) ALT as a marker for liver function, and K) Scr as a marker for kidney function. Data in (B–K) are presented as mean + SD, *n* = 3.

In addition to the antithrombogenic performance of long‐term blood‐contacting biomedical devices, it is also essential to examine their effects on blood composition and the functionality of organs such as liver and kidney. Therefore, an additional ex vivo perfusion experiment was carried out using bare and SPPIA BSA&LZM (mass ratio of 2:2) modified SR catheters to assess their potential impacts on biochemical and physiological blood parameters, including inflammatory response, blood coagulation, and organ functions (**Figure** **6**A). Note that the SR catheters utilized in this ex vivo perfusion experiment are longer than those in ex vivo blood circulation experiment for ensuring adequate contact between blood and catheter surfaces. As depicted in Figure 6B–D, no evidence of a higher tendency for blood clotting was observed for both the SR and SPPIA BSA&LZM groups, where the activated partial thromboplastin time (APTT), prothrombin fragment 1+2 (F1+2), and platelet count (PLT) exhibited minimal changes during circulation. As a foreign body, the implanted devices could be inevitably monitored by the immune system, potentially leading to acute local or even systemic inflammation reactions. However, the inflammatory parameters, including the count of complement component 3a (C3a, C3 cleavage fragment indicating activation of the classic or alternative complement pathway), c‐reactive protein (CRP, an acute‐phase protein for measuring acute inflammation), white blood cells (WBC), interleukin 10 (IL‐10, an inflammatory and immunosuppressive cytokine), and tumor necrosis factor‐alpha (TNF‐α, an inflammatory and immunostimulatory cytokine), exhibited no marked changes, suggesting an absence of an inflammatory response in both groups (Figure 6E–I). Furthermore, the blood concentrations of the alanine aminotransferase (ALT) and serum creatinine (Scr) demonstrated both the SR and SPPIA BSA&LZM modified surfaces presented no evidence of liver and kidney toxicity during circulation (Figure 6J,K), confirming their biosafety.

### 2.5 Durability of SPPIA BSA&LZM Coating

**Figure 7:**
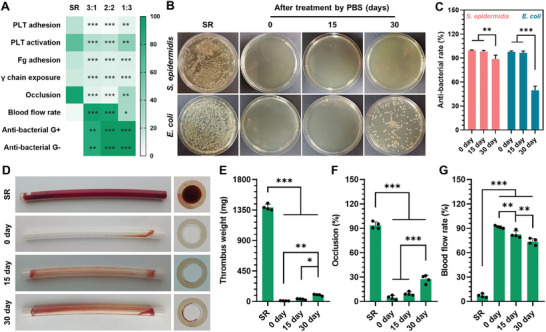
The durability of the antibacterial and antithrombogenic properties of the SPPIA BSA&LZM coating. A) Heat map of antibacterial and anticoagulation efficacy of SPPIA BSA&LZM coatings prepared with different mass ratios of BSA to LZM. B) Typical colonies of *S. epidermidis* and *E. coli* after 24 h incubation on the SPPIA BSA&LZM coated SR substrate after 15 and 30 days preimmersion in PBS. C) Quantitative analysis of the antibacterial rate against *S. epidermidis* and *E. coli*, calculated from colony counts (*n* = 4, mean + SD). D) Photographs of side and cross‐sectional views of pre‐immersed catheters after 2 h circulation. E) Thrombus weight, F) occlusion ratio, and G) blood flow rate of pre‐immersed catheters after 2 h circulation (*n* = 4, mean + SD). Statistical analysis was performed using one‐way ANOVA with ^*^ *p* < 0.05, ^**^ *p* < 0.01 and ^***^ *p* < 0.001.

The long‐term durability of surface functionality is essential for indwelling biomedical devices. Therefore, we tested the durability of the SPPIA BSA&LZM coating by immersion in PBS for periods of 15 and 30 days. Following PBS treatment, the in vitro antibacterial and ex vivo blood circulation experiments were performed. The optimal SPPIA BSA&LZM coating, with a 2:2 mass ratio of BSA to LZM, was selected for the durability tests due to its promising performance in antithrombotic and antibacterial experiments (**Figure** **7**A). As depicted in Figure 7B, our SPPIA BSA&LZM coating maintained excellent antibacterial efficacy after 15 days of immersion, with antibacterial rates against *S. epidermidis* and *E. coli* remaining above 95%. However, a notable decline in antibacterial rate was observed after 30 days of immersion. This decrease is likely due to the release of electrostatically assembled proteins from the coating. Specifically, the antibacterial rate against *S. epidermidis* dropped to 88.58%, while for *E. coli*, it reduced to only 49.63% (Figure 7C). It has been reported that the bactericidal effect of LZM is more potent against Gram‐positive bacteria.^[^ ^28^ ^]^ Thus, as the LZM content diminishes over time, the antibacterial efficacy of the SPPIA BSA&LZM coating tends to diminish first against Gram‐negative bacteria, such as *E. coli*. Similarly, the antithrombotic efficacy of SPPIA BSA&LZM coating also decreased over time. The cross‐sectional photograph revealed that more clotting was formed on the inner wall of catheters with the increase in immersion time (Figure 7D). The weight of thrombus formed on SPPIA BSA&LZM coating increased more than ten‐fold after 30 days of immersion (Figure 7E), suggesting the weakened antithrombotic efficacy of SPPIA BSA&LZM coating. Such a conclusion can be further confirmed by the quantitative analysis of occlusion ratio and blood flow rate (Figure 7F,G). Notably, despite the decrease in antibacterial and antithrombotic efficacy, our SPPIA BSA&LZM coating still performed superior to the SR group.

## 3 Conclusion

In this study, we proposed a facile SPPIA method for fabricating active dual‐protein coating with high preservation of protein activity and medium‐term stability for the application of CVCs to suppress thrombosis and infection. This strategy was realized first by the partial disruption of S─S bonds in BSA and LZM using the oxidant SP, leading to the formation of particle‐like nucleation platforms through hydrophobic interactions among proteins. Subsequently, due to the opposite surface charges of the residual nonoxidized BSA and LZM under neutral conditions, the self‐assembly of proteins was initiated by the electrostatic interactions. This process promoted the growth of nucleation platforms and their inter‐particle fusion on the surfaces of substrates, forming an active dual‐protein coating. The resulting coating exhibited excellent antithrombogenic properties, which are mainly attributed to the bioinert nature of BSA and negatively charged ─SO_3_H groups formed during protein oxidization. Additionally, the electrostatic assembly process effectively preserved the activity of LZM, endowing the coating with potent bactericidal properties against both *S. epidermidis* and *E. coli*. Notably, this coating retained the resistance against thrombosis and bacteria even after 15 days of PBS exposure. Overall, our stepwise assembly approach not only provides a new strategy for the fabrication of active protein coatings but also shows promise for the surface engineering technology of blood‐contacting catheters

## 4 Experimental Section

### Materials

BSA, LZM (from egg white), SP, PBS, ANS, ThT, FITC‐PEG‐succinimidyl easter (FITC‐PEG‐NHS), and Rhodamine‐PEG‐succinimidyl easter (Rh‐PEG‐NHS) were purchased from Aladdin Bio‐Chem Technology Co., Ltd. (Shanghai, China). HUVECs were purchased from Jennio Biotech Co., Ltd. (Guangzhou, China). Cell counting kit‐8 (CCK‐8) was provided by DOJINDO Co., Ltd. (Shanghai, China). The Si wafer was provided by Kejing Material Technology Co., Ltd. (Hefei, China). LIVE/DEAD BacLight bacterial viability kit was provided by ThermoFisher Co., Ltd. (Shanghai, China). The SR wafer and catheter were purchased from Ledger General Hygiene Materials Co., Ltd. (Changzhou, China).

### Preparation of SPPIA BSA&LZM

To analyze the mechanism of coating formation, SPPIA BSA&LZM was prepared in an aqueous solution containing BSA (1 mg mL^−1^), LZM (1 mg mL^−1^), and SP (1 mg mL^−1^). The reaction times varied and are specified in the respective experimental sections. For coating deposition, various mass ratios of BSA to LZM (3:1, 2:2, and 1:3) were employed, with a constant SP concentration of 1 mg mL^−1^ and a total BSA&LZM concentration of 2 mg mL^−1^. Before coating, SR wafers and catheters were subjected to oxygen plasma treatment for 10 min to enhance coating adhesion. These substrates were then dipped into the prepared solution for 6 h at 37 °C, followed by ultrasonically cleaning with distilled water. The deposition process was repeated thrice to ensure complete coating coverage. For Si wafers, a single deposition process was performed for coating formation analysis. After deposition, the samples were dried by nitrogen gas and stored for further use. Partially oxidized BSA and LZM were prepared following the same protocol in aqueous solution, with concentrations of 2 mg mL^−1^ for both BSA and LZM.

### Far‐UV CD Assay

The CD spectra of SPPIA BSA&LZM with different mass ratios after 6 h reaction in the aqueous solution were analyzed using a J‐1500 Spectrometer (Jasco, Japan). Spectra were recorded in the range of 190 to 260 nm at 1 nm intervals. Native BSA&LZM was included for comparative analysis as a control. The quantitation of secondary structural content was performed using the analysis software provided by the spectrometer manufacturer.

### ANS Binding Assay

ANS was employed as a fluorescent probe to evaluate the exposure of hydrophobic residues in SPPIA BSA&LZM. Native BSA&LZM with different mass ratios were incubated with ANS (200 µm) for 30 min in the dark. Then, an equivalent volume of SP (2 mg mL^−1^) was added. After 6 h reaction, the fluorescence intensity of ANS was quantified using an FLS‐920 fluorescence spectrophotometer (Edinburgh Instruments). The emission spectra were recorded in the range of 410 to 590 nm with the excitation wavelength set at 355 nm. The mixture of native BSA&LZM and ANS without adding SP was set as control.

### ThT Fluorescence

The presence of amyloid structure in SPPIA BSA&LZM was confirmed by the ThT staining. Briefly, the Si substrates coated with SPPIA BSA&LZM at varying mass ratios were immersed in the ThT solution (100 µm). For the control group of native BSA&LZM, the protein solution was dropped on the substrates and dried in a vacuum. Then, the samples were analyzed using a fluorescence spectrophotometer, with spectra recorded in the range of 450 to 600 nm and an excitation wavelength set at 440 nm.

### Characteristics of SPPIA BSA&LZM Coatings

The chemical structures of SPPIA BSA&LZM coatings were characterized using FTIR (Nicolet 5700, USA). X‐ray photoelectron spectroscopy (XPS) analysis was performed on a Thermo Scientific K‐Alpha Spectrometer (USA) equipped with a monochromatic Al Kα X‐ray source (1486.6 eV) and operated at 100 W. The samples were examined under vacuum (*p* < 10^−8^ mbar) with survey scans at a pass energy of 150 eV and high‐resolution scans at 25 eV. XPS data were processed and analyzed using Avantage software. The particle size distribution of the partially oxidized BSA, LZM, and SPPIA BSA&LZM after 5 and 30 min of reaction was determined by Dynamic Light Scattering (DLS) using a Malvern Zeta sizer Nano‐ZS90. The inter‐particle fusion on the sample surface was assessed by measuring surface morphology with a color 3D laser scanning microscope (VK‐X3000, KEYENCE, Japan). WCA measurements were conducted using a DSA100 drop shape analysis system (KRuSS GmbH Company, Hamburg, Germany). The macroscopic and microscopic surface morphology were observed using a camera (Z7II, Nikon Ltd., Japan) and SEM (JSM‐7001F, JEOL Ltd., Japan), respectively. An Elliptic Polarization Spectrometer (M‐2000 V, J.A. Woollam, USA) was employed to determine the coating thickness using the Cauchy model.

### Integration of BSA&LZM in Coatings

Before coating, BSA and LZM were incubated with FITC‐PEG‐NHS and Rh‐PEG‐NHS, respectively. This mixture was stored in the dark overnight. After incubation, the reaction mixture was dialyzed against distilled water using a 10 kDa dialysis bag to remove unbound fluorescent markers. Then, the marked proteins were employed for coating deposition following the protocol mentioned above. Note that the deposition process was conducted only once to preserve the fluorescent intensity of the proteins. After coating, the sample surfaces were examined using a laser confocal microscope with excitation wavelengths of 488 and 560 nm.

### Antibacterial Assay

The antibacterial properties of SPPIA coatings were evaluated in accordance with the ISO22196‐2011 guidelines.^[^ ^33^ ^]^ Gram‐positive *S. epidermidis* (strain ATCC 6538) and Gram‐negative *E. coli* (strain ATCC 25 922) were utilized in the antibacterial tests. First, monoclonal colonies were precultured for 12 h and then diluted to a concentration of 6 × 10^5^ cells mL^−1^. Bare and SPPIA BSA&LZM coated SR substrates were subjected to UV sterilization before being placed in sterile petri dishes. Then, 100 µL of the diluted bacterial solution was evenly spread on the sample surfaces by gently covering a polyethylene film. The samples were incubated at 37 °C and a humidity level of no less than 90% for 24 h. After incubation, the samples were gently rinsed with saline for the observation by SEM. For antibacterial rate assessment, the incubated samples were rinsed with saline, then inverted on agar and cultured in the aforementioned conditions for another 24 h. The antibacterial rate was calculated using the following formula:

(1) $$R=\left(N_{C}-N\right)/N_{C}\times 100\%$$

where N_C_ and N represent the number of colonies in the control and experimental groups, respectively.

To further determine the bactericidal effect of SPPIA BSA&LZM coatings, a solution culture method was performed. Bacteria were incubated on the bare and SPPIA BSA&LZM coated SR substrates, following the same process as in the solid culture method. After incubation, the samples were rinsed with 1 mL of saline, and 500 µL of this solution was added to 5 mL of liquid culture medium, followed by incubation at 37 °C for 12 h. Finally, 200 µL of the solution was taken out to measure the OD value at 600 nm. The antibacterial rate was calculated using the following formula:

(2) $$R=\left[\left(OD_{C}-\mathrm{OD}\right)/\left(OD_{C}-OD_{M}\right)\right]\times 100\%$$

where OD_C_, OD, and OD_M_ represent the OD values of the control, the sample, and the liquid culture medium, respectively.

### Animal Ethics

All animal experiments were conducted following the guidelines of the Council for the Purpose of Control and Supervision of Experiments on Animals, Ministry of Public Health, China. These experiments received approval from The Dongguan People's Hospital Laboratory Animal Welfare and Ethics Committee (Approval No. IACUC‐AWEC‐202302003).

### Cell and Blood Compatibility

For the cell compatibility assessment, HUVECs were cultured in DMEM/F12 medium supplemented with 20% FBS and seeded on the samples at a density of 2 × 10^4^ cells cm^−2^. After 24 and 72 h of incubation at 37 °C with 5% CO_2_ in a humidified incubator, the cell viability was evaluated using a CCK‐8 assay. For the hemolysis test, fresh rabbit blood (anticoagulated with 3.8% sodium citrate) was diluted with saline (for samples and negative control group) or ultrapure water (for positive control group) at a ratio of 4:5. The samples were incubated with 200 µL of the diluted blood at 37 °C for 1 h. After that, the supernatant was collected, and its absorbance at 540 nm was measured using an enzyme reader. The hemolysis rate was calculated using the following formula:

(3) $$\mathrm{Hemolysis}\left(\%\right)=\left[\left(A-C\right)/\left(B-C\right)\right]\times 100\%$$

where A, B, and C represent the absorbance value of samples, the positive control group, and the negative control group, respectively.

### Fg Adsorption and Conformational Change Assay

PPP was prepared by centrifuging the fresh rabbit blood at 3000 rpm for 15 min. Then, the samples were incubated with 50 µL of PPP at 37 °C for 2 h, followed by blocking with 1 mg mL^−1^ BSA. For the Fg adsorption test, the samples were incubated with Fg monoclonal antibody (diluted 1:1000 in PBS) at 37 °C for 1 h. Meanwhile, for the activity assessment of the Fg γ chain, samples were incubated with Fg γ chain monoclonal antibody (diluted 1:200 in PBS) at 37 °C for 1 h. After incubation, all the samples were thoroughly rinsed with PBS and incubated with HRP‐labeled polyclonal IgG antibodies. The colorimetric reaction was initiated using 3,3′,5,5′‐tetramethylbenzidine (TMB) for 10 min and terminated with an acid solution. The absorbance of the resulting solution was measured at 405 nm.

### Platelet Adhesion and Activation Assay

PRP was obtained by centrifuging the fresh rabbit blood at 1500 rpm for 15 min. The samples were then incubated with 50 µL of PRP at 37 °C for 30 min. After incubation, the samples were rinsed thrice with saline and fixed in 2.5% glutaraldehyde solution overnight. The adherent platelets on the sample surfaces were dehydrated and dealcoholized, then observed using SEM. The number of adhered and activated platelets was counted from four SEM images.

### Ex Vivo Thrombogenicity Test

The detailed experimental procedure has been previously described.^[^ ^34^ ^]^ In brief, SR catheters before and after coating with SPPIA BSA&LZM were connected to the left carotid artery and the right jugular vein of rabbits, forming a closed loop for blood circulation. After 2 h of circulation, the catheters were collected and rinsed with saline. Both side‐view and cross‐sectional images of the catheters were captured. The occlusion ratio was determined by calculating the ratio of the thrombus area to the internal area of the catheters based on the cross‐sectional images. In addition, the thrombus formed on the luminal surface were weighed and their morphology was examined by SEM.

### Blood Analysis by Ex Vivo Blood Circulation

The whole blood was collected at various time points (0, 5, 30, and 60 min) during the ex vivo blood circulation. To more closely mimic practical conditions, prolonged catheters (length 1.6 meters, inner diameter 3 mm) were used to enlarge the contact area between the catheter and the blood. The collected blood samples were submitted to the Blood Analysis Centre of Dongguan People's Hospital for hematological and blood biochemistry analysis, including WBC, PLT, ALT, Scr, APTT, F1+2, C3a, CRP, IL‐10, and TNF‐α.

### Long‐Term Stability of SPPIA BSA&LZM Coating

The durability of the SPPIA BSA&LZM coating was evaluated through a long‐term stability test. The coated samples were preimmersed in PBS solution for 15 and 30 days, with the solution being replaced every 7 days. After immersion, the antibacterial and ex vivo blood circulation were performed.

### Statistical Analysis

The data were presented as the mean ± standard deviation. All experiments were independently repeated four times (*n* = 4) unless specified otherwise. Statistical differences between groups were evaluated using one‐way ANOVA. The analysis was performed using GraphPad PRISM 9.0 software (GraphPad Software, Inc., San Diego, US). Statistical significance was defined as ^*^ *p* < 0.05, ^**^ *p* < 0.01, and ^***^ *p* < 0.001 for all experiments.

## Conflict of Interest

The authors declare no conflict of interest.

## Supporting information

Supporting Information

## Data Availability

The data that support the findings of this study are available from the corresponding author upon reasonable request.
