# Supplementary material for: Active Dual‐Protein Coating Assisted by Stepwise Protein–Protein Interactions Assembly Reduces Thrombosis and Infection
Source: Adv Sci (Weinh). 2024 Feb 29;11(17):2310259. doi: 10.1002/advs.202310259 (PMC11077678; doi:10.1002/advs.202310259)
Supplement: Supplementary file 1 — Supporting Information [file ADVS-11-2310259-s001.pdf]

ADVANCED SCIENCE
Open Access

Supporting Information

for *Adv. Sci.*, DOI 10.1002/advs.202310259

Active Dual-Protein Coating Assisted by Stepwise Protein–Protein Interactions Assembly Reduces Thrombosis and Infection

Wentai Zhang, Jiangling Zhang, Fangkun Hu, Wenxuan Wang, Zeyu Du, You Ke, Qing Ma, Xiaohui Mou, Jing Lu* and Zhilu Yang**

Supporting Information

Active Dual-Protein Coating Assisted by Stepwise Protein-Protein Interactions Assembly Reduces Thrombosis and Infection

Wentai Zhang,[a] Jiangling Zhang,[b] Fangkun Hu,[b] Wenxuan Wang,[b] Zeyu Du,[a,b] You Ke,[a,b] Qing Ma,[a,b] Xiaohui Mou,[a,b,] Jing Lu,[c,]* and Zhilu Yang[a,]**

[a] Dongguan Key Laboratory of Smart Biomaterials and Regenerative Medicine, The Tenth Affiliated Hospital, Southern Medical University, Dongguan, Guangdong, 523000, China.
E-mail address:

[b] School of Materials Science and Engineering, Key Lab of Advanced Technology for Materials of Education Ministry, Southwest Jiaotong University, Chengdu 610031, China.
E-mail address:

[c] Department of Anesthesiology, Sichuan Provincial People's Hospital, University of Electronic Science and Technology of China, Chengdu, Sichuan, 610072, China.

Corresponding authors e-mail address: xiaohuimou0313@163.com (X. Mou); lujing.1979@hotmail.com (J. Lu); zhiluyang1029@smu.edu.cn (Z. Yang)

Animal Ethics Statement

All animal experiments were conducted following the guidelines of the Council for the Purpose of Control and Supervision of Experiments on Animals, Ministry of Public Health, China. These experiments received approval from The Dongguan People's Hospital Laboratory Animal Welfare and Ethics Committee (Approval No. IACUC-AWEC-202302003).

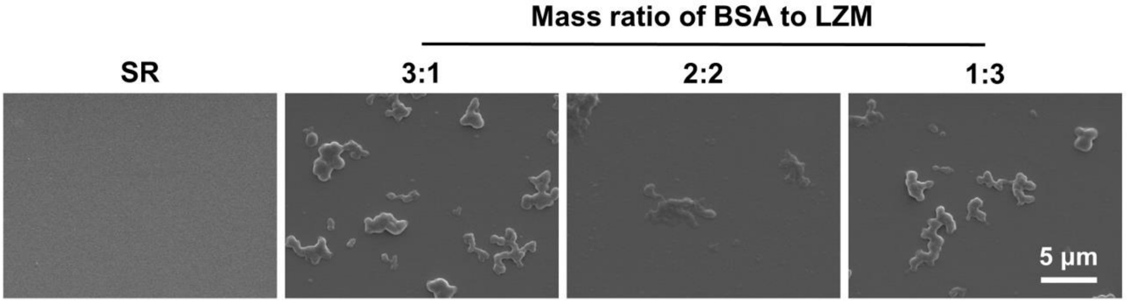

Figure S1. Surface morphology of bare and SPPIA BSA&LZM coated SR substrates prepared with different mass ratios of BSA to LZM.

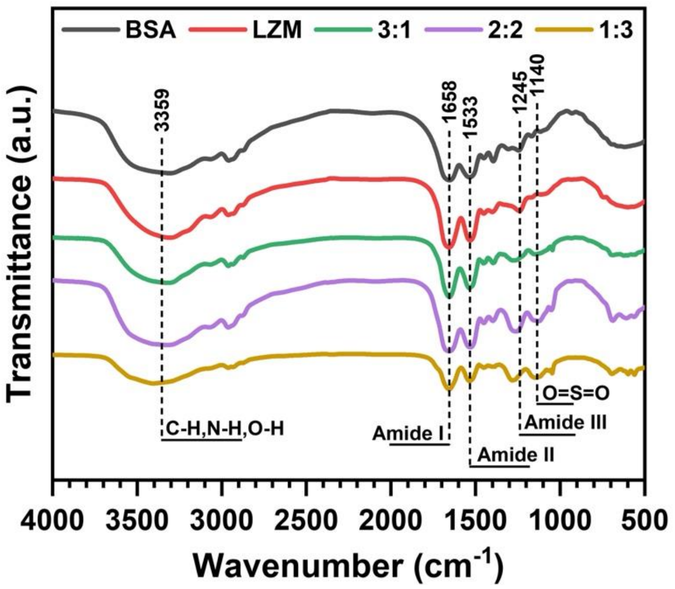

Figure S2. FTIR spectra of native BSA, native LZM, and SPPIA BSA&LZM with different mass ratios of BSA to LZM.

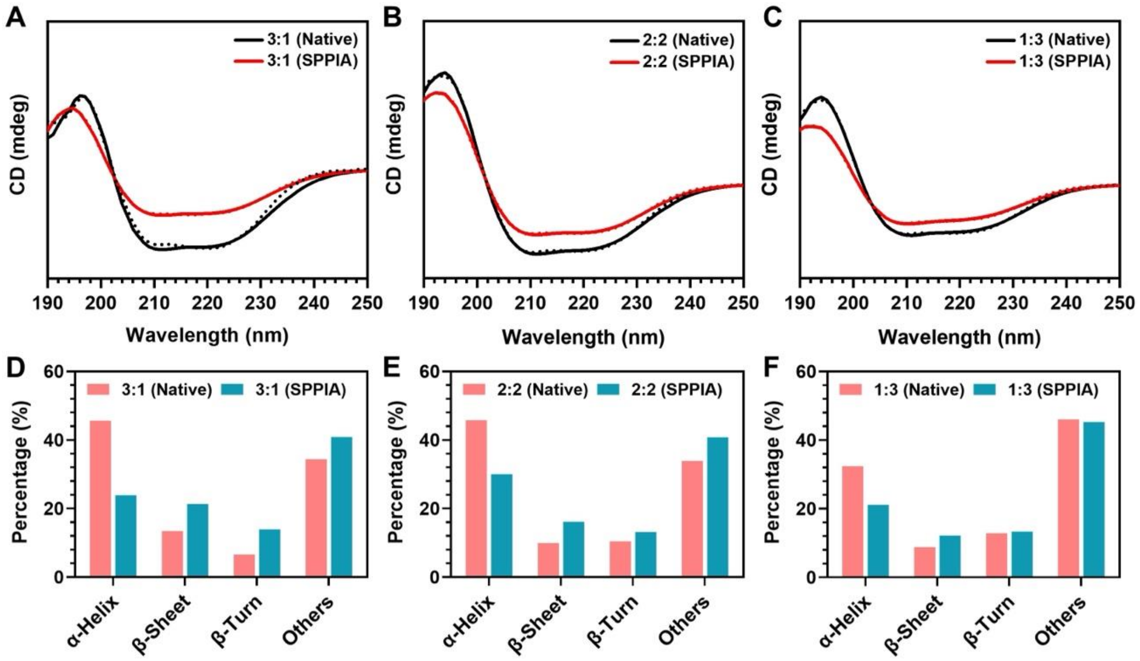

Figure S3. (A-C) CD spectra of SPPIA BSA&LZM with different mass ratios of BSA to LZM. (D-F) Quantitation of structural content in the SPPIA BSA&LZM calculated by the CD analysis.

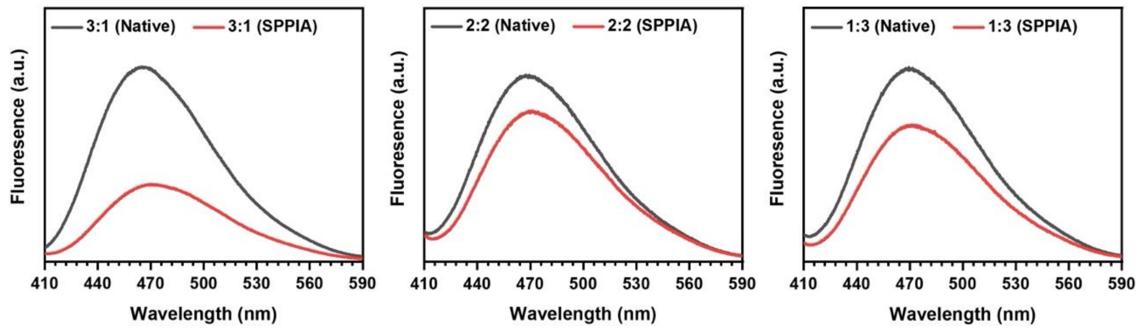

Figure S4. ANS fluorescence spectra of SPPIA BSA&LZM prepared with different mass ratios of BSA to LZM.

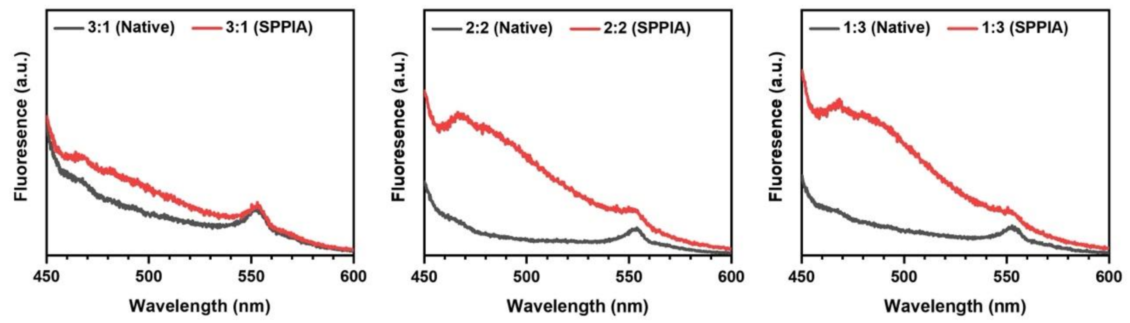

Figure S5. ThT fluorescence spectra of SPPIA BSA&LZM prepared with different mass ratios of BSA to LZM.

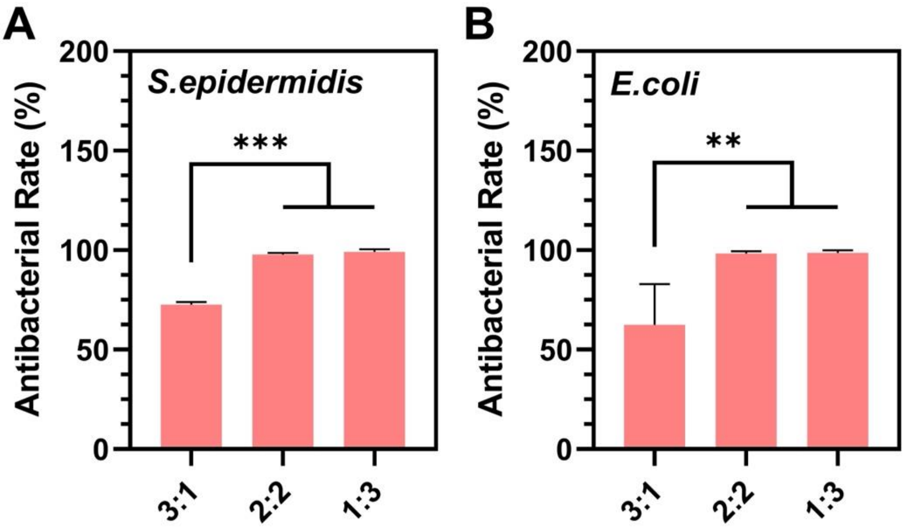

Figure S6. Anti-bacterial rate of SPPIA BSA&LZM coating against (A) *S. epidermidis* and (B) *E. coil* calculated by the optical density (OD) value. Data are presented as mean + SD, n=4. Statistical analysis was performed using one-way ANOVA with $^{**}P < 0.01$ and $^{***}P < 0.001$.

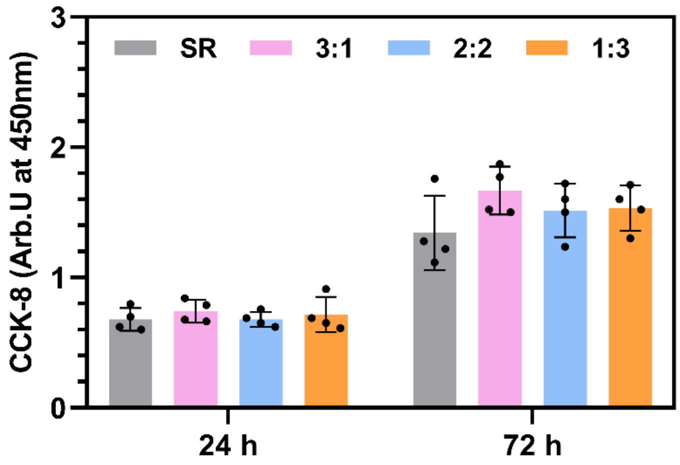

Figure S7. Viability of human umbilical vein endothelial cells (HUVECs) after 24 and 72 h incubation in the extracts of SPPIA BSA&LZM coated SR substrates. Data are presented as mean ± SD, n=4.

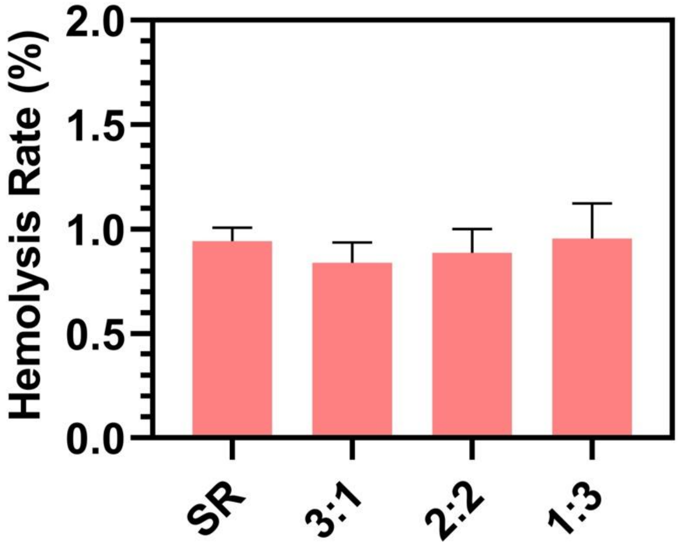

Figure S8. Hemolysis rate of SPPIA BSA&LZM coated SR substrates prepared with different mass ratios of BSA to LZM. Data are presented as mean ± SD, n=4
